# CDCA2 promotes the proliferation of colorectal cancer cells by activating the AKT/CCND1 pathway in vitro and in vivo

**DOI:** 10.1186/s12885-019-5793-z

**Published:** 2019-06-13

**Authors:** Yifei Feng, Wenwei Qian, Yue Zhang, Wen Peng, Jie Li, Qiou Gu, Dongjian Ji, Zhiyuan Zhang, Qingyuan Wang, Dongsheng Zhang, Yueming Sun

**Affiliations:** 1 The First School of Clinical Medicine, Nanjing Medical University, Nanjing, Jiangsu China; 2 Department of General Surgery, the First Affiliated Hospital of Nanjing Medical University, 300 Guangzhou Road, Nanjing, 210029 Jiangsu China; 3 Department of General Surgery, Jinling Clinical Medical College, The Affiliated Hospital of Southeast University, Nanjing, Jiangsu 210002 People’s Republic of China

**Keywords:** CDCA2, CCND1, Proliferation, PI3K/AKT pathway, Colorectal cancer

## Abstract

**Background:**

Cell division cycle associated 2 (CDCA2), upregulated in lung adenocarcinoma and oral squamous cell carcinoma, may be related to some malignant diseases. Nevertheless, its role in colorectal cancer (CRC) remains unknown.

**Methods:**

CDCA2 expression was analyzed using The Cancer Genome Atlas (TCGA), quantitative real-time PCR (qRT-PCR), and immunohistochemistry. The impact of CDCA2 on cell proliferation was analyzed via loss- or gain-of-function assays. Furthermore, gene set enrichment analysis was conducted to explore the potential mechanism of CDCA2 in CRC. Lastly, the expression levels of CCND1 and AKT were measured in CRC cell lines.

**Results:**

Our study revealed that CDCA2 expression was associated with tumor progression. Through loss- or gain-of-function assays, we found that upregulation of CDCA2 promoted the proliferation of DLD-1 cells, however, downregulation of CDCA2 in SW480 cells restrained proliferative capacity both in vitro and in vivo*.* The results of flow cytometry showed that CDCA2 promoted cell cycle progression via upregulation of CCND1 in CRC cell lines. In the following experiments, we found that CDCA2 regulated CCND1 expression through activating the PI3K/AKT pathway, and confirmed this using a specific PI3K inhibitor (LY294002).

**Conclusions:**

This study demonstrates that overexpression of CDCA2 might target CCND1 to promote CRC cell proliferation and tumorigenesis through activation of the PI3K/AKT pathway.

## Background

Colorectal cancer (CRC) is one of the most common malignancies worldwide [1, 2] with high incidence and death rates in China [3, 4]. In spite of the increasing attention gained by CRC, the multistep process by which CRC develops remains unclear due to the complex molecules involved [5]. Therefore, more studies are required to understand the molecular mechanisms involved in tumor formation and progression, and facilitate effective diagnosis and treatment of CRC.

Cell division cycle associated 2 (CDCA2) was found to be a cell cycle-related protein whose expression was correlated with several other proteins, such as CDCA1, 3, and 4–8 [6]. Several recent studies have found that CDCA2 can regulate the expression of PP1γ-dependent essential DNA damage response [7, 8] in the cell cycle and preserve the characteristic chromosome architecture for the transition to interphase [7]. Moreover, CDCA2 modulates the phosphorylation of the major mitotic histone H3 in a PP1-dependent manner [9]. An increasing number of reports have shown that CDCA2, which is upregulated in neuroblastoma [10], oral squamous cell carcinoma tissue [11], and lung adenocarcinoma [12], may be related to certain malignant diseases. Nevertheless, the relationship between CDCA2 and CRC remains to be elucidated. The purpose of this study was to detect the exact role of CDCA2 in CRC. Through suppressing or upregulating the expression of CDCA2, we showed the functional and clinical results of a comprehensive analysis for aberrant expression of CDCA2 in CRC.

## Methods

### Tissue samples and cell lines

A total of 120 CRC and 115 adjacent non-tumor colorectal specimens were obtained from Jiangsu Province Hospital between June 2014 and June 2016. The Research Ethics Committee of Nanjing Medical University has approved the research, and we obtained written informed consent from all patients. All the samples were obtained surgically and conserved at − 80 °C. Patients were not included in this study if they received any preoperative treatment. For the in vitro experiments, cell lines, including five types of CRC cells (SW480, LoVo, DLD-1, HCT116, HT29) and a intestinal mucosal epithelial cell (NCM460), all were conserved in the laboratory. The cell culture medium consisted mostly of Dulbecco’s modified Eagle’s medium (DMEM), including 100 U/mL penicillin, 100 μg/mL streptomycin and 10% fetal bovine serum (Wisent, Canada). All cells were cultured in a 5% CO2 atmosphere at about 37 °C. We purchased the PI3K inhibitor LY294002 from Cell Signaling Technology (Danvers, MA, USA) and the inhibitor was used to treat CRC cells at 10 μM.

### Immunohistochemical (IHC) analysis

IHC was conducted to detect protein expression of CDCA2 in 30 CRC tissues and 25 non-tumor tissues. IHC staining, which was performed according to standard immunoperoxidase staining procedure, was independently reviewed by two experienced pathologists. The staining intensity score was calculated as follows: 0, negative; 1, weak; 2, moderate; and 3, strong. The percentage of positive cells was calculated as follows: 0, negative; 1, < 33%;2, 34–66%; 3, > 67%. The final scores were based on the sum of these two scores, scored as follows: -(total score 0); + (total score 1 and 2); ++ (total score 3 and 4) and +++ (total score 5 and 6).

### RNA extraction and qRT-PCR assay

Total RNA was extracted using a TRIzol extraction kit (Invitrogen, Carlsbad, CA, USA) followed by the manufacturer’s protocol. The qRT-PCR assay was carried out by means of a PCR kit (Roche Diagnostics, Indianapolis, IN, USA). Subsequently, the final step was conducted with the StepOnePlus Real-time System (Applied Biosystems, Foster City, CA, USA). The gene-specific primer sequences were as follows: CDCA2: forward 5′-TGCCGAATTACCTCCTAATCCT-3′ and reverse 5′- TGCTCTACGGTTACTGTGGAAA-3′, p21: forward 5′- TGTCCGTCAGAACCCATGC-3′ and reverse 5′- AAAGTCGAAGTTCCATCGCTC-3′, p27: forward 5′- AGGAGGAGATAGAAGCGCAGA-3′ and reverse 5′- GTGCGGACTTGGTACAGGT-3′, CCND1: forward 5′- GCTGCGAAGTGGAAACCATC-3′ and reverse 5′- CCTCCTTCTGCACACATTTGAA-3′, CCNB1: forward 5′- AATAAGGCGAAGATCAACATGGC-3′ and reverse 5′- TTTGTTACCAATGTCCCCAAGAG-3′, CCNE1: forward 5′- AAGGAGCGGGACACCATGA-3′ and reverse 5′- ACGGTCACGTTTGCCTTCC-3′, CDK2: forward 5′- CCAGGAGTTACTTCTATGCCTGA-3′ and reverse 5′- TTCATCCAGGGGAGGTACAAC-3′, GAPDH: forward 5′- GGAGCGAGATCCCTCCAAAAT-3′ and reverse 5′- GGCTGTTGTCATACTTCTCATGG-3′. The 2^–ΔΔCt^ method was used to analyzed the data. All qRT-PCR processes were carried out in triplicate.

### Data of patients and samples from the Cancer genome atlas

We downloaded normalized RNA expression data (level 3) from the RNASeq v2 system, which were provided by the Cancer Genome Atlas project (TCGA) (https://portal.gdc.cancer.gov/). We defined the exclusion criteria: i) histological results excluded a CRC tissue; ii) patients had other malignant diseases at the same time; iii) samples lack of necessary data; and iv) patients receiving neoadjuvant therapy. At last, 351 tumor tissues and 32 normal tissues were used in this study.

### Knockdown and overexpression of CDCA2

Negative control (NC) and small interfering RNAs (siRNAs) of CDCA2 were constructed by GenePharma Corporation (Shanghai, China). The siRNA sequences of CDCA2 were as follows: siRNA1, 5′-CACCUGCCUUUCUAAAUAUTT-3′; siRNA2, 5′-GGGCAAAGGAUCAAGUGAUTT-3′; siRNA3, 5′-CUGCCUUGGAAAGGAUUGATT-3′. Transfection was performed with Lipofectamine 3000 (Invitrogen) following the manufacturer’s instructions. The assays were performed 48 h after transfection to assess the knockdown efficiency. A CDCA2 inhibitor lentivirus (shCDCA2) was then constructed according to siRNA1. To upregulate the expression of CDCA2 in DLD-1 cells, mammalian expression plasmids (pReceiver-M02-CDCA2) designed to specifically express CDCA2 were obtained from GeneCopoeia (Rockville, MD, USA).

### Colony formation assay

Five hundred cells were cultured in the wells of six-well plates 48 h after transfection. Two weeks later, each well of the plates was bathed with cold phosphate-buffered saline (PBS) 2–3 times, soaked in 95% alcohol for about 30 s, then dyed with crystal violet for 10 min. Afterwards, a Nikon light microscope (Nikon Corporation, Tokyo, Japan) were employed to count the spots (≥50 cells/spot). And a Canon digital camera (Canon DS126211, Inc., Tokyo, Japan) were used to capture images.

### Cell proliferation assay

The Cell Counting Kit-8 (CCK-8; Dojindo, Tokyo, Japan) assay was used to detect cell viability. Firstly, 96-well plates were seeded with 2 × 10^3^ cells. Then, 100 μL reagent, 10% of which was CCK-8 reagent, was supplemented to each well 24, 48, 72, and 96 h later. Two hours later, We measured the absorbance at a test wavelength (450 nm) and a reference wavelength (630 nm) by using a microplate reader.

### 5-Ethynyl-2′-deoxyuridine assay

5-Ethynyl-2′-deoxyuridine (EdU) assay kit (RiboBio, China, C10310–3) was used to measure cell proliferation. Briefly, before the addition of EdU (50 μM), cells were seeded into 24-well plates (2 × 10^4^ cells/well) and cultured with DMEM for 24 h. The cells were then immersed in formaldehyde (4%) for 30 min and soaked in 0.5% Triton X-100 for 10 min. 400 μL 1× ApolloR reaction cocktail was then supplemented. After a 30 min reaction, to bring out the nuclei, Hoechest 33,342 (400 μL) was added. 30 min later, images of the cells were shot by a Nikon microscope (Nikon, Japan). To assess cell proliferation, we then randomly selected three fields and calculated the mean number of cells.

### Flow cytometric analysis

The cells were diposed by trypsin and centrifuged for 5 min at 1200 rpm. The cells were firstly washed with PBS 2–3 times and then soaked in 75% ethanol before saved at − 20 °C overnight. After being washed twice with PBS and incubated with RNAse, the cells were stained with PI staining solution (500 μL) for about 15 min at room temperature. PI (10 μg/mL; Sigma-Aldrich) and Annexin V-FITC (50 μg/mL, BD Biosciences) were used to incubate with the apoptotic cells in dark place for about 15 min. The data were acquired using a FACScan flow cytometer (BD Biosciences, Franklin Lakes, NJ, USA).

### Western blotting

Followed by the manufacturer’s guidelines, protein lysates from the different cells were treated with a RIPA kit (Beyotime, Shanghai, China). Before transferring to polyvinylidene difluoride membranes (Millipore, Bedford, MA, USA), the different-weight proteins would be separated on 10% sodium dodecyl sulfate-polyacrylamide gels in running buffer. The membranes were soaked in 5% skim milk for 2 h at room temperature and then soaked in primary antibodies at 4 °C overnight. The membranes were then incubated with anti-mouse or anti-rabbit IgG for 2 h at room temperature and then washed with TBST buffer three times. The bands were exposed by ECL Plus (Millipore, Billerica, MA, USA) in a Bio-Imaging System. The antibodies included were as follows: CDCA2 (1:1000, no. ab45129), CCND1 (1:10000, no.ab134175), CCNE1 (1:1000, no.ab33911), CCNB1 (1:25000, no. ab32053), p-AKT (1:500, no.ab38449), AKT (1:500, no.ab8805), anti-rabbit secondary antibodies (1:5000, no. GAB007), and anti-mouse secondary antibodies (1:5000, no. GAM007). We used GAPDH (1:5000, no.ab8245) as a control.

### In vivo assay

We purchased totally 12 male mice (3–5 weeks, 12–16 g) from the Laboratory Animal Center. We then randomly divided the mice into two groups (shNC and shCDCA2) and injected 2 × 10^6^ CRC cells (shCDCA2 or shNC SW480 cells) subcutaneously per rat. We measured bi-dimensional tumor extent every 4 days. Four weeks later, all the mice were executed by dislocation of the cervical vertebra and all implanted tumors were surgically collected. The formula to figure up the tumor volume was as follows: volume = (width^2^ × length)/2. All the experiments above were carried out followed by the protocols of the NJMU Institutional Animal Care and Use Committee.

### Statistical analysis

We used the Statistical Program for Social Sciences 20.0 software (SPSS, CA, USA) and GraphPad Prism 5.0 (GraphPad Software, CA, USA) to analyze the data. Chi-square test (Table 1) was used to analyze the clinical features. The Wilcoxon rank-sum test was used in Table 2. Pearson’s correlation test was carried out to examine the relationship between CDCA2 and Ki-67. One-way analysis of variance or student’s *t*-test were used to analyze the treated and control groups. A *P*-value < 0.05 indicated statistical significance.

**Table 1:** Relationship between CDCA2 expression and clinicopathological characteristics of CRC patients (*n* = 90)

Characteristics	No.	Expression of CDCA2		*P*-value
		Low expression (*n* = 45)	High expression (*n* = 45)	
Age (years)				
<60	28	15	13	0.649
≥60	62	30	32	
Sex				
Male	51	23	28	0.288
Female	39	22	17	
Tumor diameter				
<5 cm	47	31	16	0.002
≥5 cm	43	14	29	
Primary tumor site				
Colon	43	19	24	0.291
Rectum	47	26	21	
Depth of invasion				
T1 + T2	26	17	9	0.063
T3 + T4	64	28	36	
Lymph node invasion				
Negative	46	29	17	0.011
Positive	44	16	28	
Distant metastasis				
Negative	75	40	35	0.157
Positive	15	5	10	
TNM stage				
I/II	44	25	19	0.206
III/IV	46	20	26

**Table 2:** Statistical analysis of CDCA2 expression in CRC and adjacent normal tissues by immunohistochemistry

	n	CDCA2 expression				*P*-value
		–	+	++	+++	
CRC tissues	30	2	9	17	2	*P* < 0.001
Adjacent normal tissue	25	12	10	3	0	

## Results

### CDCA2 was overexpressed in CRC cells and correlated with advanced clinical factors of CRC

**Fig. 1:**
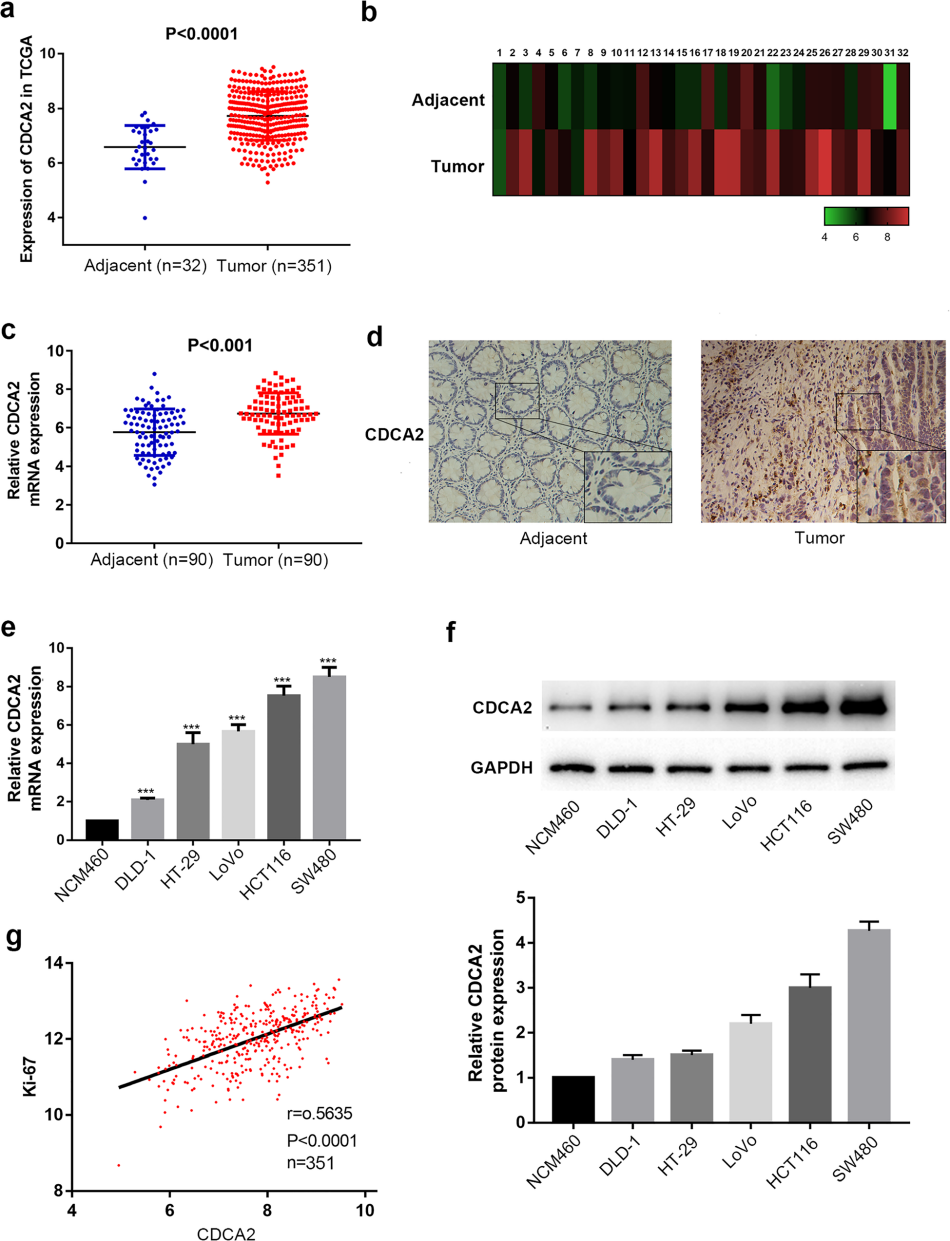
Expression of CDCA2 in CRC tissue and cells. **a** Expression of CDCA2 was frequently upregulated in 32 adjacent non-tumor tissue samples (Adjacent) compared with 337 colorectal tumor tissues (Tumor) in the TCGA profile. **b** CDCA2 expression was markedly increased in 32 paired CRC tissue samples in the TCGA profile. **c** Relative mRNA expression of CDCA2 in 90 pairs of CRC tissues and adjacent tissues detected by RT-PCR. **d** CDCA2 protein in 30 CRC tissues and 25 adjacent normal specimens were detected by IHC. **e** and **f** CDCA2 expression in six cell lines were detected using RT-PCR and western blotting. **g** CDCA2 expression was positively correlated with Ki-67 expression (*r* = 0.5635, *P* < 0.001). *represents *p* < 0.05, ** represents *p* < 0.01, *** represents *p* < 0.001, the data are expressed as the mean ± SD

To detect the functional role of CDCA2, we analyzed CDCA2 expression in published profiles from TCGA (https://cancergenome.nih.gov/) and found that it was overexpressed in tumor sample (337 cases) compared with normal tissues (32 cases) (*P* < 0.001; Fig. 1a). Next, we analyzed CDCA2 expression in a total of 32 paired CRC tissues in this dataset to further confirm the above result and found that it was significantly upregulated in tumor samples (*P* < 0.001; Fig. 1b). To further detect the potential role of CDCA2, we compared the level of CDCA2 mRNA expression between 90 pairs of CRC and adjacent normal tissues using qRT-PCR assay, normalized it to GAPDH (*P* < 0.001; Fig. 1c), and investigate the connection between the level of CDCA2 expression and the clinical factors of CRC. According to the median CDCA2 expression, we classified the 90 CRC cases into two groups: the high CDCA2 expression group (*n* = 45) and the low CDCA2 expression (*n* = 45) (Table 1). Obviously, high CDCA2 expression was significantly related to larger tumor size (*P* = 0.002) and lymph node invasion (*P* = 0.011). However, the expression of CDCA2 was not associated with other clinical features, like age (*P* = 0.649) and primary tumor site (*P* = 0.294). We then detected its expression using IHC staining in 55 paraffin-embedded tissues, including 25 normal tissues and 30 CRC tissues. Table 2 and Fig. 1d showed that protein expression of CDCA2 was obviously overexpressed in cancerous samples compared to non-tumor tissues. Furthermore, CDCA2 expression were raised in all five cancer cell lines compared to NCM460 cells in both protein and mRNA levels (Fig. 1e and f). Moreover, through analyzing the data from TCGA, we found that expression of CDCA2 was positively associated with expression of Ki-67 (*r* = 0.5635, *P* < 0.001) (Fig. 1g). Taken together, all of the above results strongly indicated that CDCA2 expression is upregulated in CRC cells and may be related to advanced clinicopathological features of CRC.

### CDCA2 modulated the proliferation of CRC cells

**Fig. 2:**
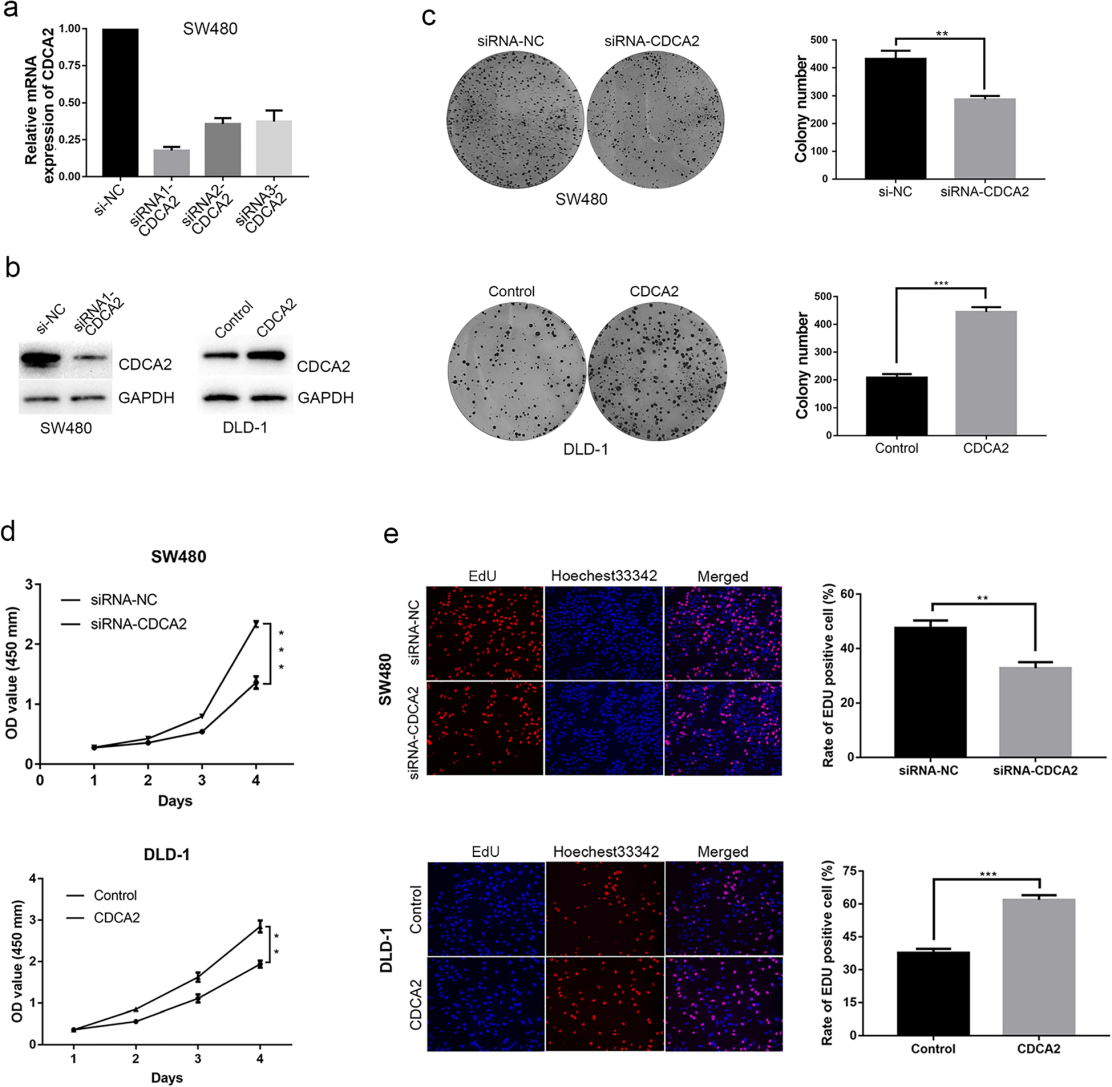
Expression of CDCA2 in CRC cells and loss or gain of function assays. **a** The levels of CDCA2 mRNA expression were verified by RT-PCR in SW480 after treatment with siRNAs against CDCA2. **b** CDCA2 protein levels were measured by western blotting. **c** Colony formation assays were performed to determine the proliferation of SW480 and DLD-1 cells. Colonies were counted and captured. **d** Effects of CDCA2 alteration on the cell viability of CRC cells were detected by CCK-8 assay. **e** EdU staining assays were performed to determine the growth of SW480 and DLD-1 cells. Representative images and data are based on three independent experiments. * represents *p* < 0.05, ** represents *p* < 0.01, *** represents *p* < 0.001

To further confirm the function of CDCA2 in CRC, SW480 cells were transiently incubated with small interfering RNAs targeting CDCA2, while CDCA2 expression was upregulated in DLD-1 cells (Fig. 1e and f). The qRT-PCR results revealed that siRNA1-CDCA2 presented a higher efficiency of interference than siRNA2-CDCA2 and siRNA3-CDCA2 (Fig. 2a). DLD-1 cells were transiently transfected with CDCA2-carrying plasmids, as well as empty vectors. The effect was determined by western blot (Fig. 2b).

Considering that CDCA2 encodes a targeting subunit of the cell-cycle associated protein [8], we speculated whether CDCA2 played a role in promoting cell proliferation. The colony formation assay indicated that groups transfected with siRNA1 presented fewer spots than siRNA-NC-transfected groups (Fig. 2c). Similarly, the results of the CCK-8 assay showed that siRNA-CDCA2-transfected CRC cells showed evidently damaged proliferative capacity (Fig. 2d). EdU (red)/Hoechst 33342 (blue) immunostaining showed the same trend that downregulation of CDCA2 significantly inhibited the proliferative capacity of CRC cells (Fig. 2e). Finally, exogenous CDCA2 expression in DLD-1 cells enhanced their proliferation (Fig. 2c, d, and e). According to the results of the three assays, we concluded that CDCA2 can promote proliferation of CRC cell lines.

### Silencing of CDCA2 inhibited tumor growth in a xenograft mouse

**Fig. 3:**
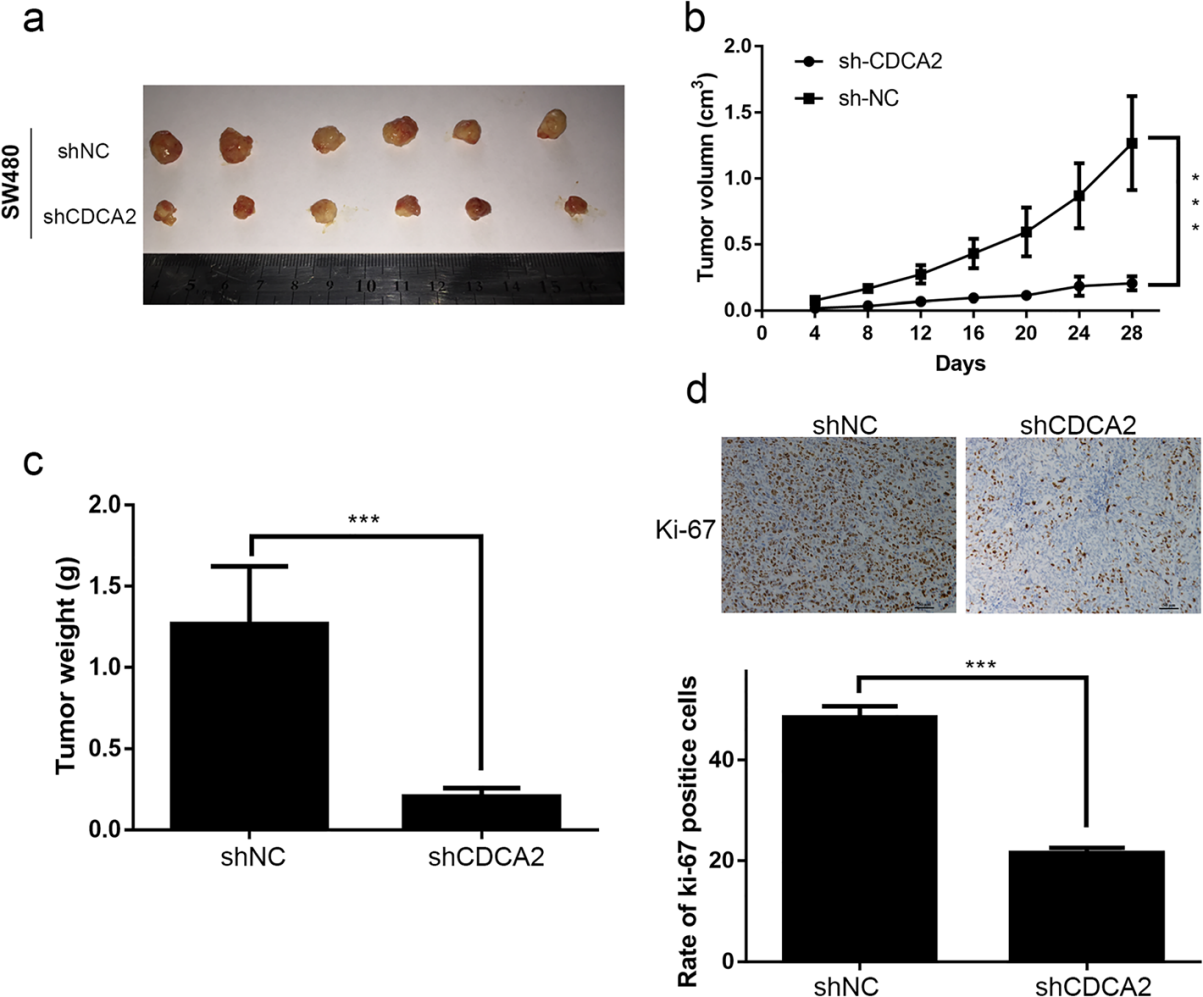
Downregulation of CDCA2 inhibits CRC tumorigenesis in nude mice. **a** The size of tumors formed by SW480 cells in the shCDCA2 and control groups. **b** and **c** Tumor volume and the weight of the tumors were analyzed. **d** Representative images of sections sliced from indicated tumors and stained with anti-Ki67

To further detect the effect of CDCA2 in tumorigenesis in nude mice, we performed tumorigenesis assay. SW480 cells were incubated with CDCA2 inhibitor lentivirus (shCDCA2) or empty vectors (shNC). Cells (2 × 10^6^) transfected with shCDCA2 or shNC were separately injected subcutaneously into nude rats. Tumor size and volume was respectively measured and calculated every 4 days. All implanted tumors were collected 28 days after injection (Fig. 3a). Figure 3b showed that tumor growth was much slower (Fig. 3b) in shCDCA2 group, which also presented lower mean weight compared with shNC group (Fig. 3c). IHC result revealed that tumors taken from shCDCA2-transfected cells presented lower intensity of Ki-67 staining compared with those derived from cells transfected with empty vector (Fig. 3d). In conclusion, these in vitro and in vivo assays demonstrated that CDCA2 may play an important role in the proliferation of CRC cells.

### CDCA2 promoted the G1/S phase transition of the CRC cell cycle by upregulating CCND1

**Fig. 4:**
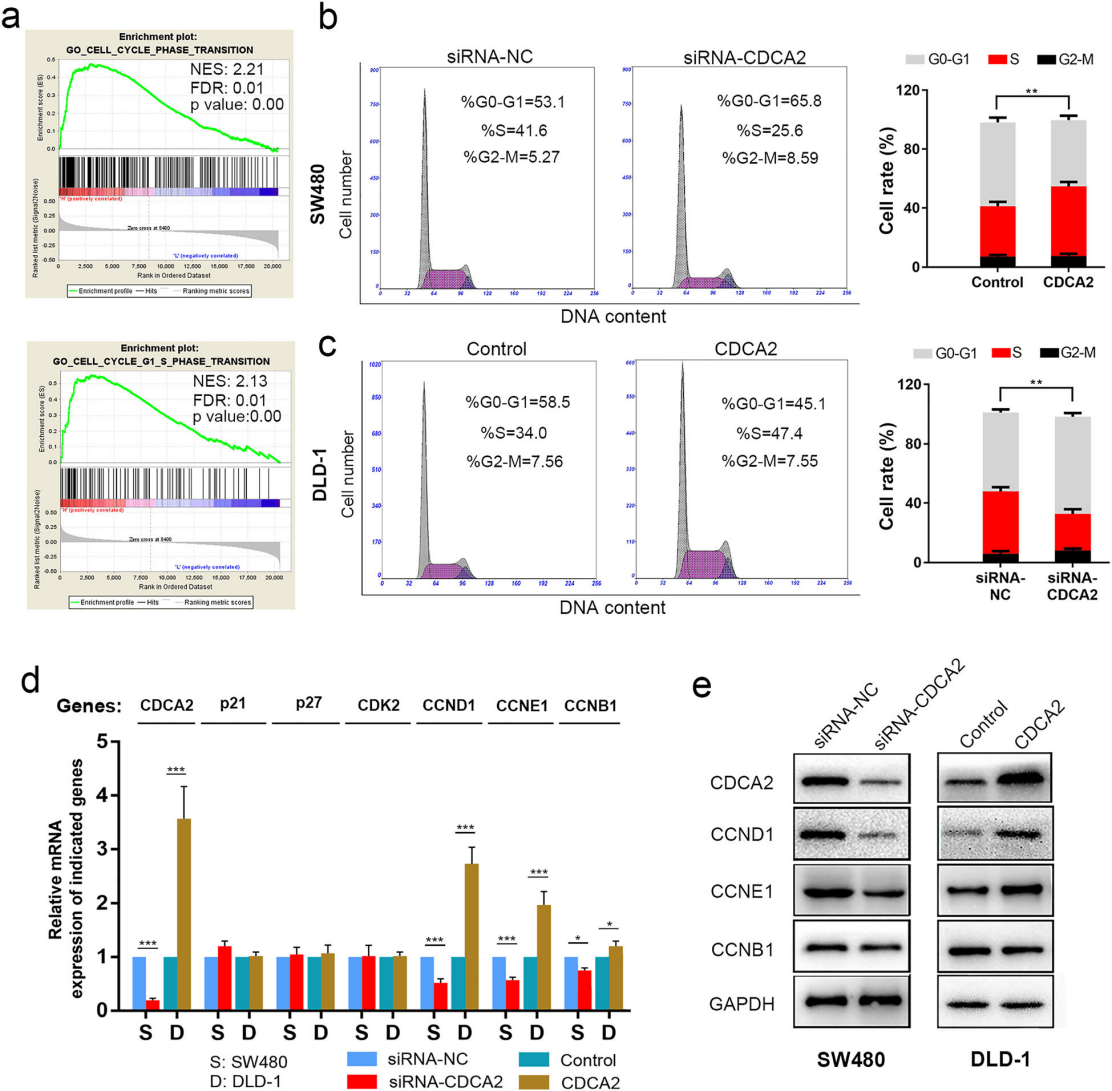
CDCA2 influences the G1/S phase transition of the cell cycle through regulating CCND1 expression. **a** GSEA plot showing that CDCA2 expression positively correlated with cell-cycle-activated gene signatures, especially in G1-S phase transition (GO_CELL_CYCLE_PHASE_TRANSITION, GO_CELL_CYCLE_G1 _S_PHASE_TRANSITION). **b** and **c** CDCA2 promoted the G1/S transition in CRC cell lines. **d** qRT-PCR was conducted to determine the key regulators of the G1-S phase transition. **e** Key regulators of the G1 phase were evaluated by western blotting to clarify the specific role of CDCA2 in the G1/S phase transition

To explore the possible mechanism by which CDCA2 influences the proliferation of CRC cells, we determined the CDCA2 expression from TCGA via gene set enrichment analysis (GSEA) [13] and found that CDCA2 expression levels were positively correlated with cell proliferation by affecting genes in the cell cycle phase transition, especially in G1-S phase transition (Fig. 4a). To verify the results of this analysis, we measured cell cycle distribution in SW480 and DLD-1 cell lines. As shown in Fig. 4b, siRNA-CDCA2 treatment induced an increase in the percentage of SW480 cells in the G1 phase compared to that by siRNA-NC treatment. Accordingly, upregulation of CDCA2 accelerated the G1/S transition in DLD-1 cells (Fig. 4c). Moreover, qRT-PCR was performed to determine the key checkpoints of the G1-S phase transition, and results revealed that knockdown of CDCA2 led to the downregulation of CCND1, CCNE1, and CCNB1 expression, whereas overexpression of CDCA2 upregulated the expression of these molecules (Fig. 4d). However, altering the expression of CDCA2 had no effect on the expression of p21, p27, and CDK2 (Fig. 4d). We further confirmed this through western blotting. The results showed that CCND1 changed most apparently following up- or down-regulation of CDCA2 (Fig. 4e). Therefore, we believed that the regulation of cell cycle by CDCA2 may be mainly achieved through CCND1.

### PI3K/AKT signaling pathway was involved in CDCA2-induced CRC cell proliferation

**Fig. 5:**
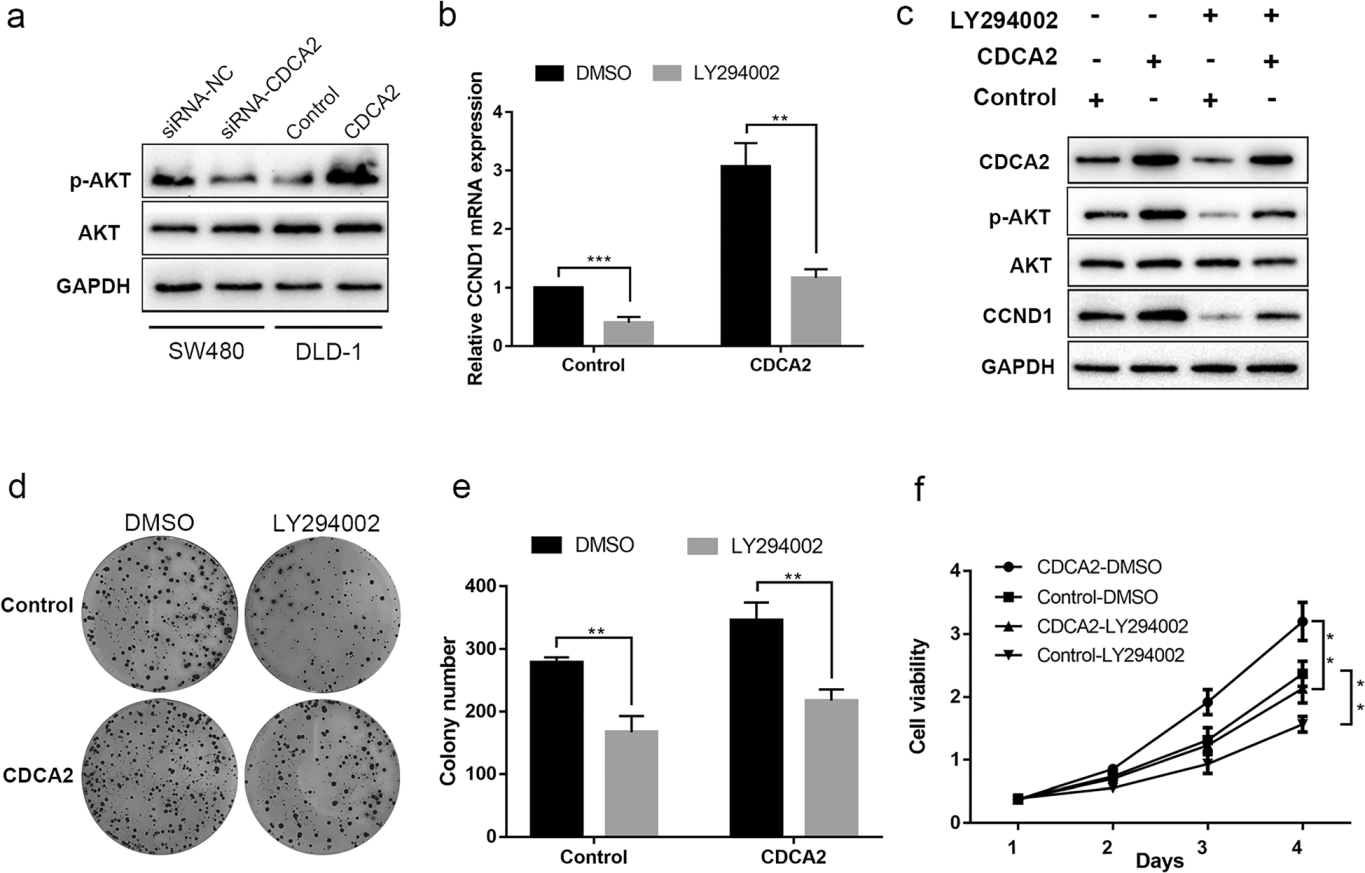
PI3K/AKT signaling pathway was involved in CDCA2-induced colon cancer cell proliferation. **a** Western blot was used to detected wheather PI3K/AKT pathway was involved in the regulation of proliferation under the influence of CDCA2. **b** CCND1 mRNA expression was detected in the indicated cell lines under the infuence of LY294002. **c** Western blotting was used to detect the relationship among CDCA2, PI3K/AKT pathway and CCND1. **d**, **e** and **f** Rescue experiments (colony formation and cck-8 rescue assays) were conducted to further confirm the above results

The PI3K/AKT pathway plays an essential role in cell proliferation in various types of cancer and is closely linked with the adjustment of CCND1 levels [14, 15]. Hence, we performed western blotting to detect whether the PI3K/AKT pathway was involved in the adjustment of proliferation under the influence of CDCA2. As shown in Fig. 5a, the expression of phosphorylated AKT in SW480 cells transfected with siRNA-CDCA2 decreased, with no change observed in the expression of total AKT. The opposite results were obtained in CDCA2-overexpressing DLD-1 cells (Fig. 5a). We therefore questioned whether the PI3K/AKT pathway participates in the regulation of CCND1 expression by CDCA2.

To verify the hypothesis, CDCA2-overexpressing DLD-1 cells were treated with a specific PI3K inhibitor (LY294002). qRT-PCR and western blot analysis were then carried out, and results showed that treatment of CDCA2-overexpressing DLD-1 cells with LY294002 for 24 h obviously inhibited p-AKT activity and promoted the decline of CCND1 expression (Fig. 5b and c), indicating that CCND1 accumulation could be suppressed by the addition of inhibitors in CRC cell lines. We then performed rescue experiments to detect whether the suppression of the PI3K/AKT pathway could reverse the proliferation-promoting effect induced by CDCA2 overexpression. Compared to DMSO treatment, LY294002 treatment significantly weakened the growth ability of CDCA2-overexpressing DLD-1 cells (Fig. 5d and e). These results implicated a role of CDCA2 in cell cycle progression through activation of the PI3K/AKT pathway in CRC.

## Discussion

In addition to previous studies, which reported that upregulation of CDCA2 is common in lung adenocarcinoma and oral squamous cell carcinoma tissue, we found that CDCA2 transcripts were upregulated in CRC tissues versus non-malignant tissues, and its level of expression was positively correlated with greater tumor size and lymph node invasion, suggesting that CDCA2 may play an important role in CRC progression. Consistently, CDCA2 expression detected in CRC cell lines showed the same trend. We then further confirmed this result through loss- or gain-of-function assays. As shown in Fig. 2, colony formation, growth rate, and DNA replication were significantly inhibited by CDCA2 knockdown in SW480 cells, indicating obvious inhibition of proliferation in vitro. Conversely, upregulation of CDCA2 induced malignant tumor cell behaviors, as indicated by the higher cell proliferation rate in CCK-8 assay, higher clonogenic survival in colony formation assay, and higher proportion of cells in the DNA replication phase in the EdU assay. The in vivo assay further confirmed the function of CDCA2. GSEA was run to explore the potential role of CDCA2 in CRC. We then measured the distribution of CRC cell cycle phases by flow cytometry. Results showed that CDCA2 played an important role in promoting cell cycle transition from G1 to S phase.

Many molecules that promote or inhibit the cell cycle are involved in mediating G1/S cell transition [16, 17]. To further detect the mechanisms that how CDCA2 regulates the G1/S transition, expression levels of some cell cycle key regulators were detected. As shown in Fig. 3, our results indicated that CCND1 expression changed most apparently following up- or down-regulation of CDCA2, suggesting that CDCA2 promoted cell cycle through mediating the upregulation of CCND1. CCND1 has been reported to be involved in many processes, such as cell cycle progress, chromosomal instability, mitochondrial function, and cellular aging [18–20].

Previous studies have demonstrated that the PI3K/AKT pathway plays a key role in modulating cell proliferation [21]. Moreover, the PI3K/AKT pathway is closely related to CCND1 in several cancers [22–25], including CRC [26]. Our results showed that downregulation of CDCA2 could decrease p-AKT expression without obvious changes in the total AKT expression level. The opposite result was observed in CDCA2-overexpressing DLD-1 cells. We then further blocked the PI3K/AKT pathway using LY294002 and found that p-AKT expression decreased, followed by the decline in CDCA2 expression, which demonstrated that CDCA2 expression could be upregulated by PI3K/AKT pathway activation. The subsequent rescue function assays further confirmed the above findings.

However, there are still many areas that remain to be explored. Firstly, there are some other pathways, such as the mTOR and Wnt/B-catenin signaling pathways [27, 28], which are related to cell proliferation and CCND1 regulation, suggesting that the PI3K/AKT pathway may not be the only pathway involved in CDCA2-CCND1 regulation. Additionally, Table 1 showed that expression of CDCA2 is significantly associated with lymph node metastasis (Table 1), studies on whether CDCA2 can promote metastasis and invasiveness in vitro and in vivo remain to be conducted.

## Conclusions

The results of this study demonstrated that CDCA2 might target CCND1 to promote CRC cell proliferation and tumorigenesis at least partially through activation of the PI3K/AKT pathway, and CDCA2 might serve as a potential prognostic and therapeutic target for CRC.

## Data Availability

The datasets used and/or analyzed during the current study are available from the corresponding author on reasonable request.

## Abbreviations

CCNB1: Cyclin B1
CCND1: Cyclin D1
CCNE1: Cyclin E1
CDCA2: Cell division cycle associated 2
CDK2: Cyclin dependent kinase 2
CRC: Colorectal cancer
mRNA: Messenger RNA
P21: Cyclin dependent kinase inhibitor 1A
p27: Proteasome 26S subunit, non-ATPase 9
qRT-PCR: Quantitive real-time PCR
